# Association of a Pathway-Specific Genetic Risk Score With Risk of Radiation-Associated Contralateral Breast Cancer

**DOI:** 10.1001/jamanetworkopen.2019.12259

**Published:** 2019-09-27

**Authors:** Gordon P. Watt, Anne S. Reiner, Susan A. Smith, Daniel O. Stram, Marinela Capanu, Kathleen E. Malone, Charles F. Lynch, Esther M. John, Julia A. Knight, Lene Mellemkjær, Leslie Bernstein, Jennifer D. Brooks, Meghan Woods, Xiaolin Liang, Robert W. Haile, Nadeem Riaz, David V. Conti, Mark Robson, David Duggan, John D. Boice, Roy E. Shore, Marc Tischkowitz, Irene Orlow, Duncan C. Thomas, Patrick Concannon, Jonine L. Bernstein

**Affiliations:** 1 Memorial Sloan Kettering Cancer Center, New York, New York; 2 Department of Radiation Physics, The University of Texas MD Anderson Cancer Center, Houston; 3 Department of Preventive Medicine, University of Southern California, Los Angeles; 4 Fred Hutchinson Cancer Research Center, Seattle, Washington; 5 Department of Epidemiology, University of Iowa, Iowa City; 6 Stanford Cancer Institute, Stanford University School of Medicine, Stanford, California; 7 Lunenfeld-Tanenbaum Research Institute, Sinai Health System, Toronto, Canada; 8 Epidemiology Division, Dalla Lana School of Public Health, University of Toronto, Toronto, Canada; 9 Danish Cancer Society Research Center, Copenhagen, Denmark; 10 Beckman Research Institute, City of Hope National Medical Center, Duarte, California; 11 Cedars-Sinai Medical Center, Los Angeles, California; 12 Translational Genomics Research Institute, An Affiliate of City of Hope, Phoenix, Arizona; 13 National Council on Radiation Protection and Measurements, Bethesda, Maryland; 14 Vanderbilt University, Nashville, Tennessee; 15 New York University School of Medicine, New York; 16 University of Cambridge, Cambridge, England; 17 Genetics Institute, University of Florida, Gainesville

## Abstract

**Importance:**

Radiation therapy for breast cancer is associated with increased risk of a second primary contralateral breast cancer, but the genetic factors modifying this association are not well understood.

**Objective:**

To determine whether a genetic risk score comprising single nucleotide polymorphisms in the nonhomologous end-joining DNA repair pathway is associated with radiation-associated contralateral breast cancer.

**Design, Setting, and Participants:**

This case-control study included a case group of women with contralateral breast cancer that was diagnosed at least 1 year after a first primary breast cancer who were individually matched to a control group of women with unilateral breast cancer. Inclusion criteria were receiving a first invasive breast cancer diagnosis prior to age 55 years between 1985 and 2008. Women were recruited through 8 population-based cancer registries in the United States, Canada, and Denmark as part of the Women’s Environment, Cancer, and Radiation Epidemiology Studies I (November 2000 to August 2004) and II (March 2010 to December 2012). Data analysis was conducted from July 2017 to August 2019.

**Exposures:**

Stray radiation dose to the contralateral breast during radiation therapy for the first breast cancer. A novel genetic risk score comprised of genetic variants in the nonhomologous end-joining DNA repair pathway was considered the potential effect modifier, dichotomized as high risk if the score was above the median of 74 and low risk if the score was at or below the median.

**Main Outcomes and Measures:**

The main outcome was risk of contralateral breast cancer associated with stray radiation dose stratified by genetic risk score, age, and latency.

**Results:**

A total of 5953 women were approached for study participation, and 3732 women (62.7%) agreed to participate. The median (range) age at first diagnosis was 46 (23-54) years. After 5 years of latency or more, among women who received the first diagnosis when they were younger than 40 years, exposure to 1.0 Gy (to convert to rad, multiply by 100) or more of stray radiation was associated with a 2-fold increased risk of contralateral breast cancer compared with women who were not exposed (rate ratio, 2.0 [95% CI, 1.1-3.6]). The risk was higher among women with a genetic risk score above the median (rate ratio, 3.0 [95% CI, 1.1-8.1]), and there was no association among women with a genetic risk score below the median (rate ratio, 1.3 [95% CI, 0.5-3.7]). Among younger women with a high genetic risk score, the attributable increased risk for contralateral breast cancer associated with stray radiation dose was 28%.

**Conclusions and Relevance:**

This study found an increased risk of contralateral breast cancer that was attributable to stray radiation exposure among women with a high genetic risk score and who received a first breast cancer diagnosis when they were younger than 40 years after 5 years or more of latency. This genetic risk score may help guide treatment and surveillance for women with breast cancer.

## Key Points

**Question:**

Is a genetic risk score comprising variants in a DNA repair pathway associated with risk of developing a second primary contralateral breast cancer among women who underwent radiation therapy for a primary breast cancer?

**Findings:**

In this case-control study including 3732 women who received a diagnosis for a first invasive local or regional breast cancer when they were younger than 55 years, a genetic risk score comprising variants in a DNA repair pathway was associated with increased risk of a subsequent radiation-associated contralateral breast cancer. Among younger women with a high genetic risk score, the attributable increased risk for contralateral breast cancer associated with stray radiation dose was 28%.

**Meaning:**

This genetic risk score may be a helpful tool to guide treatment for young women with breast cancer.

## Teaser

This case-control study assesses the association of a genetic risk score based on variants in a DNA repair pathway with risk of a second primary contralateral breast cancer after radiation therapy for a first breast cancer.

## Introduction

Survivors of invasive breast cancer have a high risk of developing asynchronous contralateral breast cancer.^1,2^ This risk is increased among women who are relatively young when they receive the first diagnosis,^3,4^ have a family history of breast cancer,^5,6^ or have high-penetrance mutations.^7,8,9^ Treatment with tamoxifen, aromatase inhibitors, or chemotherapy is associated with reduced risk of contralateral breast cancer,^10,11,12,13^ while stray radiation doses received to the contralateral breast during radiation therapy of the first primary tumor are associated with increased risk.^14^ Although radiation therapy is an effective cancer treatment, stray radiation during radiation therapy produces potentially carcinogenic DNA damage in unaffected tissue. Radiation exposure induces a variety of lesions in DNA, the most dangerous of which are DNA double strand breaks. In humans, double strand breaks are repaired primarily by the relatively error-prone nonhomologous end-joining (NHEJ) pathway, whereas the less error-prone homologous recombination pathway is active mainly during the G2 phase of actively cycling cells.^15^ Research has shown that mutations in DNA damage response genes are associated with hypersensitivity to ionizing radiation and a high incidence of cancer.^16^ Additionally, a 2007 study^17^ among Taiwanese women found that variants in 2 genes within the NHEJ pathway were associated with breast cancer risk. As breast cancer is the most common malignant neoplasm among women in the United States^18^ and radiation therapy is used to treat more than half of women with breast cancer,^19^ identifying risk factors for radiation-associated contralateral breast cancer is an important issue.

To date, only rare, high-penetrance genetic mutations have been evaluated in radiation-associated contralateral breast cancer, to our knowledge.^7,20,21,22^ A recent genome-wide association study of survivors of childhood cancers treated with radiation therapy identified several loci that may interact with radiation exposure to increase risk of subsequent breast cancer,^23^ but it is uncertain whether risk of cancer after radiation therapy among children can be generalized to risk of cancer after radiation therapy among adults. Genetic risk scores (GRSs), which aggregate the associations of many genetic variants, are a promising method to identify associations that are not detectable for individual variants.^24^ Therefore, we genotyped single nucleotide polymorphisms (SNPs) in women with contralateral breast cancer (case group) and individually matched women with unilateral breast cancer (control group) in the Women’s Environmental Cancer and Radiation Epidemiology (WECARE) Study to develop a GRS that captures variation in risk of contralateral breast cancer associated with stray radiation exposure. We considered several approaches to develop this GRS, including aggregating SNPs associated with contralateral breast cancer in the WECARE Study, aggregating SNPs associated with contralateral breast cancer in the literature,^24^ and aggregating SNPs located in or near genes in specific DNA damage response pathways.

In this study, we focused on a GRS comprising genes in the NHEJ DNA damage response pathway, which is the most common pathway for DNA double strand break response in humans in all phases of the cell cycle. The rationale for focusing on the NHEJ pathway is that genetic variation in genes involved in DNA damage response is associated with cancer risk^16,20^; we hypothesized that having a greater number of alleles associated with cancer risk in the NHEJ pathway would be associated with a greater risk of contralateral breast cancer associated with exposure to stray radiation. By aggregating SNPs in and adjacent to the 7 genes encoding the NHEJ pathway, we assessed whether a high score on this pathway-specific NHEJ GRS was associated with a high risk of contralateral breast cancer subsequent to stray radiation exposure in the WECARE Study.

## Methods

### Study Population

The WECARE Study is a multicenter, population-based, case-control study of women with contralateral breast cancer as the case group and individually-matched women with unilateral breast cancer as the control group. Participants were recruited through 8 population-based cancer registries in the United States, Canada, and Denmark in 2 phases: the WECARE Study I,^25^ which recruited from November 2000 to August 2004, and the WECARE Study II,^26^ which recruited from March 2010 to December 2012. Participants included women who had a first invasive local or regional breast cancer that was diagnosed between 1985 and 2008 and a subsequent primary invasive cancer or carcinoma in situ in the contralateral breast (≥1 year after the first primary diagnosis for the WECARE Study I,^25^ and ≥2 years after the first primary diagnosis in the WECARE Study II^26^) with no cancers in the intervening period. Breast cancer primary status was further confirmed by the Surveillance, Epidemiology, and End Results program for participants in the United States and by medical record review after identification in the Danish Cancer Registry for women in Denmark. The control group included women who had received a first primary local or regional breast cancer and no subsequent contralateral breast cancer. Women in the control group were individually matched with women in the case group at a ratio of 2 to 1 in the WECARE Study I^25^ and a ratio of 1 to 1 in the WECARE Study II^26^ by age when they received the first breast cancer diagnosis (5-year strata), diagnosis year (4-year strata), cancer registry region, and self-reported race/ethnicity. The at-risk period for a woman with contralateral breast cancer was the time between when she received the first breast cancer diagnosis to when she received the second breast cancer diagnoses. Women in the control group were randomly selected from women in the risk set who were living and had not been diagnosed with any cancer during the interval after they received the first diagnosis corresponding to the length of the at-risk period of the matching woman with contralateral breast cancer. In the WECARE Study I,^25^ case-control sets were counter-matched such that exactly 2 members of the triad had cancer registry–reported radiation treatment for the primary breast cancer, which increased power to detect gene-environment interactions for contralateral breast cancer. A summary of enrollment criteria for each phase of the WECARE Study is provided in eTable 1 in the Supplement.

The study was approved at each participating research center by its institutional review board and, in Denmark, additionally by the ethics committee system. Participants gave verbal informed consent for a telephone interview that collected sociodemographic and clinical data, including a detailed breast cancer risk-factor history. After obtaining written informed consent, biological specimens were collected for DNA extraction and genotyping (peripheral blood cells in WECARE Study I^25^ and saliva in WECARE Study II^26^). Data on tumor characteristics and treatment were obtained from cancer registries and by abstraction of medical records. We followed the Strengthening the Reporting of Observational Studies in Epidemiology (STROBE) reporting guideline.

### Quantification of Radiation Dose to the Contralateral Breast

Radiation dose estimation methods have been detailed previously.^25^ Briefly, radiation dose to the contralateral breast was quantified for 4 quadrants (ie, upper left, upper right, lower left, lower right) and the areola region. The dose estimates were based on individual radiation parameters (eg, field locations and sizes, energy of the radiation beam, and radiation therapy dose delivered) from available information, including radiation therapy records, summary notes, and abstracted physician correspondence from medical records. Stray doses to the contralateral breast quadrants and areola were measured using lithium fluoride powder thermoluminescent dosimeters placed in tissue-equivalent phantoms, molded on women in treatment position. All reported doses are location-specific to estimate the radiation dose received by the affected quadrants or areola of the woman’s contralateral breast during treatment for the first breast cancer and the corresponding region in the unaffected breast of her matched control, as previously described.^14^

### SNP Genotyping

In the WECARE Study I,^25^ SNPs were genotyped using the HumanOmni1-Quad BeadChip platform (Illumina). In the WECARE Study II,^26^ SNP genotyping was conducted using 2 custom oligonucleotide probe panels using the Infinium iSelect HD Custom BeadChip (Illumina). Quality control methods have been detailed previously.^24^ Self-reported race/ethnicity was confirmed by principal components analysis. To reduce the probability of confounding by population stratification, final analyses using genetic data were restricted to non-Hispanic white women.

### Development of the NHEJ GRS

The NHEJ GRS is comprised of SNPs in or near the 7 genes in the NHEJ pathway: *DCLRE1C* (OMIM 602450), *LIG4* (OMIM 606593), *NHEJ1* (OMIM 611291), *PRKDC* (OMIM 600899), *XRCC4* (OMIM 194363), *XRCC5* (OMIM 194364), and *XRCC6* (OMIM 152690). Ninety-three SNPs in the pathway passed quality control, and 24 SNPs were excluded owing to strong linkage disequilibrium (*r*^2^ > 0.5) with others in the pathway. To our knowledge, there are no reported significant associations of SNPs in the NHEJ pathway with radiation-associated second cancers. Therefore, for the final 69 SNPs, we determined the alleles associated with contralateral breast cancer risk by the directionality of the main association with contralateral breast cancer risk among all women in the WECARE Study. We chose to use the direction of association from the entire study population rather than only among those exposed to radiation to ensure that the NHEJ GRS was independent of the interaction between the score and radiation exposure. The final NHEJ GRS is the sum of risk alleles (0, 1, or 2) across all 69 NHEJ SNPs. Women in the case group and the control group were classified as high NHEJ GRS if their score was greater than the median score of the all participants, 74 (range, 57-93) alleles, and low NHEJ GRS if their score was less than or equal to the median score.

### Statistical Analysis

Multivariable-adjusted rate ratios (RRs) and corresponding 95% CIs were estimated using conditional logistic regression. Women in the control group were sampled from the failure time risk sets of the women in the case group, so the RR estimates are equivalent to those obtained in a proportional hazards model of cohort data.^27^ To account for the counter-matched WECARE Study I^25^ design, statistical models included a log-weight offset term. The WECARE Study II^26^ participants, who were matched in pairs without counter-matching on radiation therapy, were assigned an offset equal to 1. All statistical models were adjusted for known and suspected contralateral breast cancer risk factors.^28^ Several important covariates had missing values for women in the case and control groups. To address missing data, we used the missing indicator method for a matched case-control study, which improves statistical efficiency relative to a complete-case analysis and is preferred to an unmatched analysis of incomplete pairs, which is vulnerable to additional confounding.^29^

First, we estimated the association of radiation exposure with contralateral breast cancer risk. Models were developed separately for radiation therapy ever vs never exposure and for location-specific dose received to the contralateral breast (categorized as 0 Gy [to convert to rads, multiply by 100], >0 to <1.0 Gy, and ≥1.0 Gy). Stratified analyses were defined a priori by age at first diagnosis (<40 years vs ≥40 years) and latency between first and second cancers (<5 years vs ≥5 years), based on previous WECARE Study results.^30,31^ For each model of radiation dose and contralateral breast cancer risk, a single stratified model was developed to obtain stratum-specific estimates while avoiding overfitting within subgroups. Statistical tests for trend were performed across the dose categories.

For the primary analysis, we examined the joint associations of the NHEJ GRS and radiation dose with contralateral breast cancer risk, fitting conditional logistic regression models with stratification as described and with additional adjustment for 3 eigenvectors obtained in principal components analysis. We estimated the population-attributable risk fraction of contralateral breast cancer associated with radiation exposure from these results.^32^

We assessed the robustness of the NHEJ GRS associations by adjusting for a GRS by Robson et al^24^ that captures common genetic variation in known breast cancer susceptibility loci. This GRS was developed by aggregating SNPs associated with first breast cancer risk and was also associated with contralateral breast cancer risk in the WECARE Study.

All analyses were performed in SAS/STAT software version 9.4 (SAS Institute). All statistical tests were 2-sided, and statistical significance was set at less than .05.

## Results

### Characteristics of the Study Population

A total of 5953 women were approached for enrollment in the WECARE Study I^25^ or WECARE Study II,^26^ of whom 3732 (63%) gave informed consent and participated. Participant characteristics are presented in Table 1. Most participants (88%) reported non-Hispanic white race/ethnicity. Location-specific dose reconstruction was not possible for 600 women (262 women in the case group and 338 women in the control group) owing to multifocal tumors or incomplete treatment or location data, leaving 3132 women available for analyses of radiation dose and contralateral breast cancer risk. For the primary analysis involving the NHEJ GRS, we excluded 278 women with genotyping quality control issues and 347 women who were not white or were Hispanic, leaving 2507 women with location-specific dose data and successful genotyping.

**Table 1.** Characteristics of Women With Contralateral Breast Cancer and Women With Unilateral Breast Cancer From the WECARE Study With Known Radiation Therapy Status

Characteristic	Women, No. (%)		*P* Value^c^
	With Contralateral Breast Cancer (n = 1521)^a^	With Unilateral Breast Cancer (n = 2211)^b^	
Age at first primary breast cancer diagnosis, y			
<40	238 (18)	384 (18)	.98
40-49	808 (53)	1180 (53)	
>50	445 (29)	648 (29)	
Age at menarche, y			
Never menstruated	3 (0)	6 (0)	.07
<13	724 (48)	964 (44)	
≥13	791 (52)	1239 (56)	
Unknown	3 (0)	2 (0)	
Full-term pregnancies, No.			
None	322 (21)	412 (19)	.002
1	271 (18)	340 (15)	
2	559 (37)	842 (38)	
3	256 (17)	387 (18)	
≥4	108 (7)	225 (10)	
Menopausal status or age at menopause^d^			
Premenopausal	1124 (74)	1675 (76)	.34
Postmenopausal, age <45 y	195 (13)	282 (12)	
Postmenopausal, age ≥45 y	194 (13)	240 (11)	
Unknown	8 (1)	14 (1)	
Histological subtype of first diagnosis			
Ductal	1205 (79)	1772 (80)	.29
Lobular	179 (12)	222 (10)	
Other	133 (9)	213 (10)	
Unknown	4 (0)	4 (0)	
Stage of first diagnosis			
Local	1061 (70)	1442 (65)	.005
Regional	448 (29)	758 (34)	
Unknown	12 (1)	11 (1)	
Estrogen receptor status of first diagnosis^e^			
Positive	797 (52)	1253 (57)	.002
Negative	467 (31)	561 (25)	
Other or unknown^e^	257 (17)	397 (18)	
Progesterone receptor status of first diagnosis^e^			
Positive	687 (45)	1082 (49)	.01
Negative	442 (29)	549 (25)	
Other or unknown^f^	392 (26)	580 (26)	
Chemotherapy for first diagnosis			
No	699 (46)	923 (42)	.01
Yes	822 (54)	1288 (58)	
Radiation therapy for first diagnosis			
WECARE Study I^g^			
No	362 (51)	266 (50)	.89
Yes	346 (49)	1133 (50)	
WECARE Study II^g^			
No	279 (34)	256 (32)	.23
Yes	534 (66)	556 (68)	
Hormone treatment for first diagnosis			
No	964 (63)	1270 (57)	.001
Yes	557 (37)	939 (42)	
Unknown	0 (0)	2 (0)	
Characteristics of second primary breast cancers			
Histological subtype of second diagnosis			
Ductal	1273 (84)	NA	NA
Lobular	145 (10)	NA	NA
Other	54 (5)	NA	NA
Unknown	7 (1)	NA	NA
Stage of second diagnosis^h^			
In situ	137 (9)	NA	NA
Local	961 (63)	NA	NA
Regional	346 (23)	NA	NA
Distant	25 (2)	NA	NA
Unknown	44 (3)	NA	NA
Estrogen receptor status of second diagnosis			
Positive	828 (54)	NA	NA
Negative	361 (24)	NA	NA
Other or unknown^f^	332 (22)	NA	NA
Progesterone receptor status of second diagnosis			
Positive	570 (38)	NA	NA
Negative	506 (33)	NA	NA
Other or unknown^f^	445 (29)	NA	NA

Abbreviations: NA, not applicable; WECARE, Women’s Environmental Cancer and Radiation Epidemiology.

^a^ Case group.

^b^ Control group.

^c^ *P* values are from tests which compare variables by case-control status.

^d^ Menopausal status was assessed 2 years prior to receiving first breast cancer diagnosis.

^e^ Refers to receptor status of the first primary breast cancer.

^f^ Includes women for whom no laboratory test was given; the test was given, and the results were unknown; or the test was given, and the results were borderline.

^g^ The WECARE Study I^25^ used a counter-matched design based on radiation therapy requiring statistical weighting of proportions. Counter-matching was not used in the WECARE Study II,^26^ so proportions are estimated separately. Comparison *P* value is calculated on weighted proportions.

^h^ For the WECARE Study I,^25^ only invasive second cancers were eligible for inclusion; for the WECARE Study II,^26^ carcinomas in situ were also eligible for inclusion.

### Overall Association of Radiation Exposure With Contralateral Breast Cancer Risk

All models were stratified by age at first diagnosis (<40 years vs ≥40 years) and latency (<5 years vs ≥5 years). Ever having received radiation therapy was associated with increased contralateral breast cancer risk among women younger than 40 years when they received the first diagnosis after a latency of 5 years or more (RR, 1.7 [95% CI, 1.1-2.6]) (Table 2). The risk of contralateral breast cancer was not associated with radiation therapy among women younger than 40 years with less than 5 years of latency (RR, 1.3 [95% CI, 0.8-2.1]) or among women who received the first breast cancer diagnosis 40 years or older with less than 5 years of latency (RR, 0.9 [95% CI, 0.7-1.2]) or with more than 5 years of latency (RR, 1.0 [95% CI, 0.8-1.2]).

**Table 2.** Exposure to Radiation Therapy for First Primary Breast Cancer, Radiation Dose to the Contralateral Breast, and Risk of Contralateral Breast Cancer in the Women’s Environmental Cancer and Radiation Epidemiology Study

Exposure to Radiation Therapy	Women, No.		Rate Ratio^c^ (95% CI)
	With Contralateral Breast Cancer^a^	With Unilateral Breast Cancer^b^	
Overall			
Never	641	522	1 [Reference]
Ever	880	1689	1.0 (0.9-1.2)
Received first breast cancer diagnosis age <40 y with <5 y latency			
Never	45	40	1 [Reference]
Ever	65	144	1.3 (0.8-2.1)
Received first breast cancer diagnosis age <40 y with ≥5 y latency			
Never	59	58	1 [Reference]
Ever	115	169	1.7 (1.1-2.6)
Received first breast cancer diagnosis age ≥40 y with <5 y latency			
Never	214	165	1 [Reference]
Ever	262	637	0.9 (0.7-1.2)
Received first breast cancer diagnosis age ≥40 y with ≥5 y latency			
Never	323	259	1 [Reference]
Ever	438	739	1.0 (0.8-1.2)

^a^ Case group.

^b^ Control group.

^c^ Adjusted for age at first diagnosis of breast cancer, age at menarche, number of full-term pregnancies, first-degree family history of breast cancer, age at menopause, chemotherapy, hormonal therapy, estrogen receptor status of the first primary diagnosis, progesterone receptor status of the first primary diagnosis, breast cancer microscopic anatomy and cancer stage. Age- and latency-stratified coefficients are estimated in a single stratified model (N = 3732).

Exposure to location-specific stray radiation dose of 1.0 Gy or more was associated with contralateral breast cancer risk among women who received their first breast cancer diagnosis when they were younger than 40 years and with 5 years or more of latency compared with those who were not exposed to stray radiation (RR, 2.0 [95% CI, 1.1-3.6]). There was a statistically significant dose trend (*P* = .03). No association was observed between location-specific stray radiation dose and contralateral breast cancer risk in the overall study population (RR, 1.1 [95% CI, 0.9-1.3]), or other age or latency subgroups (Table 3).

**Table 3.** Radiation Dose to the Contralateral Breast During Treatment for First Primary Breast Cancer and Risk of Contralateral Breast Cancer in the Women’s Environmental Cancer and Radiation Epidemiology Study

Dose, Gy^a^	Women, No.		Rate Ratio (95% CI)^d^	*P* Value^e^
	With Contralateral Breast Cancer^b^	With Unilateral Breast Cancer^c^		
Overall				
0	542	452	1 [Reference]	.49
>0 to 1.0	388	770	1.1 (0.9-1.3)	
≥1.0	329	651	1.1 (0.9-1.3)	
Received first breast cancer diagnosis age <40 y with <5 y latency				
0	37	35	1 [Reference]	.18
>0 to 1.0	27	68	1.0 (0.5-2.0)	
≥1.0	26	54	1.6 (0.8-3.1)	
Received first breast cancer diagnosis age <40 y with ≥5 y latency				
0	48	46	1 [Reference]	.03
>0 to 1.0	43	81	1.3 (0.7-2.3)	
≥1.0	48	56	2.0 (1.1-3.6)	
Received first breast cancer diagnosis age ≥40 y with <5 y latency				
0	176	144	1 [Reference]	.98
>0 to 1.0	122	301	1.0 (0.7-1.3)	
≥1.0	95	234	1.0 (0.7-1.4)	
Received first breast cancer diagnosis age ≥40 y with ≥5 y latency				
0	281	227	1 [Reference]	.52
>0 to 1.0	196	320	1.1 (0.9-1.5)	
≥1.0	160	307	0.8 (0.6-1.1)	

SI conversion factor: To convert dose to rads, multiply by 100.

^a^ Dose refers to stray dose to the contralateral breast incurred during radiation therapy for first primary breast cancer, estimated for the location of the contralateral breast cancer for women with contralateral breast cancer and the corresponding location for the matched women with unilateral breast cancer. Age- and latency-stratified coefficients are estimated in a single stratified model (N = 3132).

^b^ Case group.

^c^ Control group.

^d^ Adjusted for age when the first diagnosis of breast cancer was received, age at menarche, number of full-term pregnancies, first-degree family history of breast cancer, age at menopause, chemotherapy, hormonal therapy, estrogen receptor status of the first primary diagnosis, progesterone receptor status of the first primary diagnosis, breast cancer microscopic anatomy, and cancer stage.

^e^ *P* value for test of trend across radiation dose categories.

### Joint Associations of NHEJ GRS and Radiation Dose on Risk of Contralateral Breast Cancer

Contralateral breast cancer risk was not associated with the interaction between individual SNPs in the NHEJ GRS and radiation dose after Bonferroni correction for multiple comparisons (corrected α-level of statistical significance = 7.3 × 10^−4^) (eTable 2 in the Supplement). The NHEJ GRS was approximately normally distributed (eFigure in the Supplement) and was dichotomized at the overall median for analysis; the median (range) GRS in the case group was 75 (57-93) alleles and the median (range) GRS in the control group was 74 (57-90) alleles, and the score was independent of radiation dose. In the high NHEJ GRS group, among women who received the first diagnosis when they were younger than 40 years with a latency of 5 years or more, a stray radiation dose of 1.0 Gy or more was associated with 3-fold greater contralateral breast cancer risk compared with no radiation exposure (RR, 3.0 [95% CI, 1.1-8.1]) (Table 4); the test for trend across dose categories was statistically significant (*P* = .03). In contrast, for women with an NHEJ GRS of 74 alleles or fewer in the same age and latency group, there was no association between radiation dose and contralateral breast cancer risk (RR, 1.3 [95% CI, 0.5-3.7]). No associations were found for women who received their first breast cancer diagnosis when they were 40 years or older. Based on these results, after a latency of 5 years or longer among women who received their first breast cancer diagnosis when they were younger than 40 years with a high NHEJ GRS, the population attributable risk fraction of contralateral breast cancer attributable to stray radiation exposure to the contralateral breast was 28%. The corresponding population attributable risk fraction among women who received their first diagnosis when they were younger than 40 years after a latency of 5 years or more with a low NHEJ GRS was 18%.

**Table 4.** NHEJ GRS, Location-Specific Radiation Dose, and Risk of Contralateral Breast Cancer in the Women’s Environmental Cancer and Radiation Epidemiology Study^a^

Group^b^	Stray Radiation Exposure, Dose, Gy	Women, No.		Rate Ratio (95% CI)^e^	*P* Value	*P* Value for Trend^f^
		With Contralateral Breast Cancer^c^	With Unilateral Breast Cancer^d^			
Received first breast cancer diagnosis age <40 y with <5 y latency						
Low NHEJ GRS	0	18	30	1 [Reference]	NA	.79
	>0 to <1.0	7	27	0.4 (0.1-1.3)	.12	
	≥1.0	16	25	1.2 (0.4-3.4)	.73	
High NHEJ GRS	0	13	12	1 [Reference]	NA	
	>0 to <1.0	10	28	0.9 (0.3-3.1)	.82	
	≥1.0	7	17	1.8 (0.5-6.1)	.35	
Received first breast cancer diagnosis age <40 y with ≥5 y latency						
Low NHEJ GRS	0	15	20	1 [Reference]	NA	.57
	>0 to <1.0	15	36	1.4 (0.5-3.8)	.57	
	≥1.0	18	27	1.3 (0.5-3.7)	.58	
High NHEJ GRS	0	21	16	1 [Reference]	NA	.03
	>0 to <1.0	15	28	1.3 (0.5-3.4)	.62	
	≥1.0	17	15	3.0 (1.1-8.1)	.03	
Received first breast cancer diagnosis age ≥40 y, <5 y latency						
Low NHEJ GRS	0	65	68	1 [Reference]	NA	.44
	>0 to <1.0	49	145	1.3 (0.8-2.1)	.34	
	≥1.0	39	110	1.2 (0.7-2.0)	.54	
High NHEJ GRS	0	76	55	1 [Reference]	NA	.69
	>0 to <1.0	49	100	1.0 (0.6-1.6)	.88	
	≥1.0	40	78	1.1 (0.6-2.0)	.69	
Received first breast cancer diagnosis age ≥40 y, ≥5 y latency						
Low NHEJ GRS	0	108	112	1 [Reference]	NA	.85
	>0 to <1.0	69	150	1.0 (0.6-1.6)	.96	
	≥1.0	67	139	1.0 (0.6-1.6)	.96	
High NHEJ GRS	0	107	71	1 [Reference]	NA	.77
	>0 to <1.0	86	110	1.3 (0.9-2.1)	.20	
	≥1.0	66	112	0.9 (0.5-1.4)	.50	

SI conversion factor: To convert dose to rads, multiply by 100.

Abbreviations: GRS, genetic risk score; NHEJ, nonhomologous end-joining.

^a^ Radiation dose refers to stray dose to the contralateral breast incurred during treatment for first primary breast cancer among women with contralateral breast cancer and estimated for the corresponding location of the contralateral breast for women with unilateral breast cancer. Age- and latency-stratified coefficients are estimated in a single stratified model (N = 2507).

^b^ GRS is divided into 2 groups based on the median (74) of risk-associated alleles for all participants. Women with 74 alleles or fewer were considered low risk. Women with 75 of more alleles were considered high risk.

^c^ Case group.

^d^ Control group.

^e^ Adjusted for age at receipt of first breast cancer diagnosis, age at menarche, number of full-term pregnancies, first-degree family history of breast cancer, age at menopause, chemotherapy, hormonal therapy, estrogen receptor status of the first primary breast cancer, progesterone receptor status of the first primary breast cancer, breast cancer histology, cancer stage, and 3 eigenvectors obtained in principal components analysis.

^f^ *P* value for test of trend across radiation dose categories.

After further adjustment for known breast cancer susceptibility variants, results were not significantly changed; the association of radiation exposure with contralateral breast cancer risk among women who received their first diagnosis when they were younger than 40 years of age after a latency of 5 years or more in the high NHEJ GRS group remained statistically significant (RR, 2.9 [95% CI, 1.0-8.0]) (eTable 3 in the Supplement). In addition, to identify genes that might be driving the overall NHEJ GRS association with contralateral breast cancer risk, we repeated the modeling for each gene individually in the high-risk subgroup (<40 years of age when they received the first diagnosis and ≥5 years latency of effect). Stratifying by the median number of risk alleles within each, we found that the associations of radiation dose with contralateral breast cancer risk varied depending on the gene analyzed (eTable 4 in the Supplement). However, for women with greater than the median number of risk alleles for any given gene within the NHEJ pathway, there was no statistically significant increased risk of contralateral breast cancer risk with exposure to radiation dose of 1.0 Gy or more.

### Post-hoc Analysis of Gene Expression in the NHEJ Pathway

To explore the mechanism whereby variation in the NHEJ GRS is associated with risk of contralateral breast cancer associated with radiation exposure, we searched the NHEJ GRS SNPs against the Genotype-Tissue Expression (GTEx) database^33^ for significant expression quantitative trait loci (eQTLs) affecting the genes encoding NHEJ components. For 5 of the 7 genes in the NHEJ pathway (*LIG4*, *NHEJ1*, *XRCC4*, *XRCC5* and *XRCC6*), we identified significant eQTLs associated with either increased or decreased expression of the corresponding NHEJ gene in multiple tissues. Notably, within each gene, significant eQTLs were always associated with a single direction of association (*i.e*., all risk alleles in *XRCC4* were associated with decreased expression and all risk alleles in *LIG4* were associated with increased expression).

## Discussion

This study found that a substantial proportion of the risk of radiation-associated contralateral breast cancer was attributable to common genetic variants in the NHEJ pathway among younger women. Our pathway-specific GRS approach, based on our a priori hypothesis that variation in a DNA damage response pathway would be associated with radiation-associated contralateral breast cancer, allowed a valid and statistically powered evaluation of genetic variation and radiation-associated contralateral breast cancer risk. Radiation-associated contralateral breast cancer risk is characterized by an inverse association with age at exposure, a latency of effect, and a proportional association with radiation dose,^14,31^ which we confirmed in the present study. We also found that women who received the first diagnosis when they were younger than 40 years and were exposed to at least 1.0 Gy of stray radiation had an increased risk of developing contralateral breast cancer after 5 years, but only if they carried many NHEJ GRS risk alleles, whereas those with a low NHEJ GRS had no significantly increased risk of contralateral breast cancer associated with radiation dose. For women who were 40 years or older when they received the first diagnosis, the risk of radiation-associated contralateral breast cancer was low regardless of latency time or NHEJ GRS. We estimated that, after a latency of 5 years or more among women with a high genetic risk who were younger than 40 years when they received the first diagnosis, 28% of the increased risk of contralateral breast cancer was attributable to stray radiation exposure. For younger women with breast cancer, these findings could influence decision-making for locoregional therapy. For example, young women with a high NHEJ GRS may consider partial-breast radiation therapy (rather than whole-breast radiation therapy when appropriate), opt for radiation therapy techniques that reduce integral dose (eg, proton-beam), or decide for non-radiation therapy–based locoregional management (eg, mastectomy).^34,35,36^ These findings may be especially important in younger women with medially located breast cancers, where the scatter dose to the contralateral breast is likely to be higher.^34,35,36^ Therefore, focusing on SNPs in the NHEJ pathway may prove useful for individualizing breast cancer treatment, particularly for women treated with radiation therapy when they are younger than 40 years.

The choice of age and latency cutoffs were based on evidence from previous studies suggesting the associations between cancer and radiation exposure are strongest for women exposed when younger than 40 years and that associations with radiation exposure are greatest after at least 5 years.^30,31^ We observed no associations between radiation exposure and contralateral breast cancer risk in the overall study population or among women who received the first diagnosis after age 40 years, affirming the importance of age- and latency-stratified analysis to identify women at increased risk of radiation-associated contralateral breast cancer.^31^

Even after accounting for known breast cancer susceptibility SNPs (eTable 3 in the Supplement), after 5 years or more of latency, women with a high NHEJ GRS who received the first diagnosis when they were younger than 40 years maintained a statistically significantly increased risk of contralateral breast cancer associated with radiation dose. The association was similar to the primary analysis, suggesting that the NHEJ GRS captures a distinct component of radiation-associated contralateral breast cancer risk that is independent of overall predisposition to contralateral breast cancer. For women undergoing treatment for their first primary breast cancer, it may be prudent to estimate both overall genetic predisposition to contralateral breast cancer and radiation-specific risk.

In our gene-by-gene analysis in the high-risk subgroup (eTable 4 in the Supplement), risk estimates for each gene were not precisely consistent with the primary NHEJ GRS results, indicating that the NHEJ GRS effectively aggregates positive but variable associations of NHEJ SNPs with radiation-associated contralateral breast cancer risk. Further, our analysis of GTEx data indicated that the SNPs comprising the NHEJ GRS include eQTLs with significant associations with the expression of NHEJ genes. The predicted direction of association with contralateral breast cancer risk was uniformly increased for the *LIG4*, *NHEJ1*, and *XRCC5* genes while uniformly decreased for the *XRCC4* and *XRCC6* genes. The consistent association of multiple NHEJ GRS risk alleles with eQTLs in a single direction suggests that the NHEJ GRS may be capturing the effect of SNP alleles on the transcription of 1 or more genes in the NHEJ pathway. This supports the hypothesis that the variation in this pathway may alter double-stranded DNA damage response, thereby increasing the risk of tumor development. However, the results from GTEx are drawn from multiple tissues that may not be appropriate proxies for breast tissue and the impact of genetic variation in the overall NHEJ pathway is likely to be complex.

### Limitations and Strengths

This study has limitations. First, although a large population of participants had quantified location-specific radiation dose, accurate dosimetry was not possible for 600 women with incomplete location or clinical data or multifocal tumors. Second, the NHEJ GRS was developed based on the directionality of SNP associations among non-Hispanic white women in the WECARE Study, which may limit generalizability to other populations. However, this does not reduce the validity of the score for this population. Third, it is not currently possible to replicate our NHEJ GRS findings in another data set, as no other study of contralateral breast cancer is large enough with the necessary biospecimens, long-term follow-up, and dosimetry and questionnaire data, to our knowledge. Strengths of the study include the large study population that allowed for subgroup analyses, individual matching to reduce confounding, and individualized dosimetry. Future studies should assess whether these results are consistent for other radiation-sensitive second primary cancers.

## Conclusions

In conclusion, this study found that as much as 28% of the risk of contralateral breast cancer was attributable to stray radiation exposure among women who received their first breast cancer diagnosis when they were younger than 40 years and who were at high genetic risk, after a latency of 5 years or more. These findings may support clinical decision-making related to radiation treatment, particularly among women for whom other modalities may be considered. The NHEJ GRS may be useful for individualizing both treatment and surveillance plans for young women who have received a first primary breast cancer diagnosis.

## Notes

Supplement. **eTable 1.** Recruitment Strategy for the WECARE Study I and WECARE Study II **eTable 2.** Association of the Interaction Between Radiation Dose And Single Nucleotide Polymorphisms in the Nonhomologous End-Joining Pathway With Contralateral Breast Cancer in the WECARE Study I and WECARE Study II **eTable 3.** Nonhomologous End-Joining Repair Genetic Risk Score, Location-Specific Dose, and Risk of Contralateral Breast Cancer Additionally Adjusting for Variation in Common Breast Cancer Susceptibility Genes in the WECARE Study **eTable 4.** Number of Risk Alleles for Each Gene in the Nonhomologous End-Joining DNA Repair Pathway, Location-Specific Radiation Dose, and Risk of Contralateral Breast Cancer in the WECARE Study Among Women Who Received Their First Breast Cancer Diagnosis When They Were Younger Than 40 Years With a Latency of 5 Years or More **eFigure.** Distribution of Nonhomologous End-Joining Genetic Risk Score in Women with Contralateral Breast Cancer and Women With Unilateral Breast Cancer in the WECARE Study
