# Supplementary material for: Association of a Pathway-Specific Genetic Risk Score With Risk of Radiation-Associated Contralateral Breast Cancer
Source: JAMA Netw Open. 2019 Sep 27;2(9):e1912259. doi: 10.1001/jamanetworkopen.2019.12259 (PMC6777239; doi:10.1001/jamanetworkopen.2019.12259)
Supplement: Supplement. — eTable 1. Recruitment Strategy for the WECARE Study I and WECARE Study II eTable 2. Association of the Interaction Between Radiation Dose And Single Nucleotide Polymorphisms in the Nonhomologous End-Joining Pathway With Contralateral Breast Cancer in the WECARE Study I and WECARE Study II eTable 3. Nonhomologous End-Joining Repair Genetic Risk Score, Location-Specific Dose, and Risk of Contralateral Breast Cancer Additionally Adjusting for Variation in Common Breast Cancer Susceptibility Genes in the WECARE Study eTable 4. Number of Risk Alleles for Each Gene in the Nonhomologous End-Joining DNA Repair Pathway, Location-Specific Radiation Dose, and Risk of Contralateral Breast Cancer in the WECARE Study Among Women Who Received Their First Breast Cancer Diagnosis When They Were Younger Than 40 Years With a Latency of 5 Years or More eFigure. Distribution of Nonhomologous End-Joining Genetic Risk Score in Women with Contralateral Breast Cancer and Women With Unilateral Breast Cancer in the WECARE Study [file jamanetwopen-2-e1912259-s001.pdf]

Supplementary Online Content

Watt GP, Reiner AS, Smith SA, et al. Association of a pathway-specific genetic risk score with risk of radiation-associated contralateral breast cancer. *JAMA Netw Open*. 2019;2(9):e1912259. doi:10.1001/jamanetworkopen.2019.12259

eTable 1. Recruitment Strategy for the WECARE Study I and WECARE Study II

eTable 2. Association of the Interaction Between Radiation Dose And Single Nucleotide Polymorphisms in the Nonhomolous End-Joining Pathway With Contralateral Breast Cancer in the WECARE Study I and WECARE Study II

eTable 3. Nonhomologous End-Joining Repair Genetic Risk Score, Location-Specific Dose, and Risk of Contralateral Breast Cancer Additionally Adjusting for Variation in Common Breast Cancer Susceptibility Genes in the WECARE Study

eTable 4. Number of Risk Alleles for Each Gene in the Nonhomologous End-Joining DNA Repair Pathway, Location-Specific Radiation Dose, and Risk of Contralateral Breast Cancer in the WECARE Study Among Women Who Received Their First Breast Cancer Diagnosis When They Were Younger Than 40 Years With a Latency of 5 Years or More

eFigure. Distribution of Nonhomologous End-Joining Genetic Risk Score in Women with Contralateral Breast Cancer and Women With Unilateral Breast Cancer in the WECARE Study

This supplementary material has been provided by the authors to give readers additional information about their work.

© 2019 Watt GP et al. *JAMA Network Open.*

eTable 1. Recruitment Strategy for the WECARE Study I and WECARE Study II

Study Characteristics	WECARE Study I[a]	WECARE Study II
Recruitment Registries	▪ The State Health Registry of Iowa<br>▪ Cancer Surveillance System of the Fred Hutchinson Cancer Research Center<br>▪ Los Angeles County Cancer Surveillance Program<br>▪ The Cancer Surveillance Program of Orange County/San Diego-Imperial Organization for Cancer Control<br>▪ The Danish Breast Cancer Cooperative Group Database supplemented by the Danish Cancer Registry	▪ The State Health Registry of Iowa<br>▪ Cancer Surveillance System of the Fred Hutchinson Cancer Research Center<br>▪ The Ontario Cancer Registry<br>▪ Greater Bay Area Cancer Registry (San Francisco Bay Area Region and Santa Clara Region)<br>▪ Sacramento and Sierra Cancer Registries (Sacramento Region)<br>▪ Los Angeles County Cancer Surveillance Program<br>▪ The Danish Breast Cancer Cooperative Group Database supplemented by the Danish Cancer Registry
Age at 1st cancer diagnosis	<55 years	
Year of 1st cancer diagnosis	1/1/1985 – 1/1/2000	1/1/1990 – 1/1/2008
Stage of 1st cancer diagnosis	local/regional	
Histology of 1st cancer diagnosis	Any	
Time between 1st and 2nd cancer diagnosis	≥ 1 year	≥ 2 years
Source of DNA for genotyping	Peripheral blood	Saliva
Stage of 2nd cancer diagnosis (Cases Only)	0 – IV	I - IV
Case:control matching	1:2 with countermatching	1:1, no countermatching

Abbreviations. WECARE, Women's Environmental Cancer and Radiation Epidemiology Study
[a] See Bernstein et al (2004) for complete methods[1]

© 2019 Watt GP et al. *JAMA Network Open.*

eTable 2. Association of the Interaction Between Radiation Dose And Single Nucleotide Polymorphisms in the Nonhomolous End-Joining Pathway With Contralateral Breast Cancer in the WECARE Study I and WECARE Study II

SNP	Gene	β[a]	SE	P-value[b]
rs1010664	*DCLRE1C*	0.02	0.27	0.95
rs4237441	*DCLRE1C*	0.01	0.22	0.96
rs7920514	*DCLRE1C*	0.04	0.10	0.67
rs11259405	*DCLRE1C*	0.14	0.08	0.09
rs11593133	*DCLRE1C*	-0.02	0.09	0.83
rs12572872	*DCLRE1C*	-0.07	0.09	0.42
rs12778197	*DCLRE1C*	0.16	0.19	0.38
rs10128350	*DCLRE1C*	0.03	0.11	0.82
rs7906967	*DCLRE1C*	0.16	0.16	0.30
rs868284	*LIG4*	0.01	0.11	0.92
rs11620361	*LIG4*	-0.07	0.11	0.51
rs10131	*LIG4*	-0.05	0.13	0.74
rs7322498	*LIG4*	0.04	0.08	0.59
rs9520823	*LIG4*	-0.02	0.09	0.81
rs3093748	*LIG4*	0.07	0.11	0.53
rs3093740	*LIG4*	-1.36	0.77	0.08
rs1151402	*LIG4*	0.00	0.08	0.97
rs9520822	*LIG4*	0.52	0.36	0.15
rs12694448	*NHEJ1*	0.09	0.08	0.29
rs359960	*NHEJ1*	-0.22	0.37	0.55
rs17574343	*NHEJ1*	-0.11	0.12	0.35
rs17608747	*NHEJ1*	0.10	0.10	0.32
rs7588654	*NHEJ1*	0.02	0.23	0.94
rs4278157	*PRKDC*	-0.05	0.20	0.81
rs4521758	*PRKDC*	0.05	0.12	0.68
rs4873772	*PRKDC*	0.15	0.09	0.08
rs1231203	*PRKDC*	-0.18	0.39	0.65
rs8178086	*PRKDC*	-0.01	0.32	0.96
rs8178074	*PRKDC*	0.04	0.19	0.85
rs8178076	*PRKDC*	0.30	0.22	0.17
rs8178068	*PRKDC*	-0.08	0.17	0.63
rs8178179	*PRKDC*	-0.18	0.19	0.36
rs2731866	*XRCC4*	-0.09	0.19	0.64
rs16900340	*XRCC4*	0.08	0.15	0.61
rs16900343	*XRCC4*	0.15	0.20	0.45
rs6869366	*XRCC4*	0.02	0.22	0.91
rs7710530	*XRCC4*	-0.05	0.14	0.70

© 2019 Watt GP et al. *JAMA Network Open.*

eTable 2 (continued). Association of the Interaction Between Radiation Dose And Single Nucleotide Polymorphisms in the Nonhomolous End-Joining Pathway With Contralateral Breast Cancer in the WECARE Study I and WECARE Study II

SNP	Gene	β[a]	SE	P-value[b]
rs17205706	*XRCC4*	-0.38	0.22	0.08
rs438554	*XRCC4*	0.28	0.18	0.13
rs958535	*XRCC4*	0.32	0.18	0.08
rs9293330	*XRCC4*	-0.02	0.08	0.81
rs6891767	*XRCC4*	-0.44	0.30	0.14
rs13178127	*XRCC4*	0.07	0.19	0.72
rs7728486	*XRCC4*	0.15	0.24	0.54
rs301275	*XRCC4*	-0.02	0.10	0.84
rs7726666	*XRCC4*	-0.02	0.21	0.90
rs2974442	*XRCC4*	0.18	0.17	0.28
rs828704	*XRCC5*	-0.07	0.10	0.47
rs16855458	*XRCC5*	0.01	0.10	0.96
rs16855489	*XRCC5*	-0.01	0.09	0.89
rs2032765	*XRCC5*	-0.01	0.14	0.95
rs207887	*XRCC5*	0.05	0.13	0.68
rs2303400	*XRCC5*	-0.01	0.09	0.89
rs16855663	*XRCC5*	-0.13	0.19	0.50
rs13006837	*XRCC5*	0.09	0.14	0.56
rs207941	*XRCC5*	0.09	0.15	0.56
rs668844	*XRCC5*	-0.02	0.08	0.78
rs207925	*XRCC5*	-0.02	0.09	0.82
rs17819382	*XRCC5*	0.19	0.29	0.52
rs10166817	*XRCC5*	-0.06	0.19	0.75
rs828702	*XRCC5*	-0.08	0.08	0.33
rs6753002	*XRCC5*	-0.07	0.10	0.50
rs13002401	*XRCC5*	-0.04	0.10	0.68
rs16855447	*XRCC5*	0.24	0.19	0.21
rs1364726	*XRCC5*	-0.14	0.13	0.27
rs207905	*XRCC5*	-0.05	0.12	0.67
rs11912946	*XRCC6*	0.34	0.34	0.32
rs11703638	*XRCC6*	0.06	0.09	0.47
rs132771	*XRCC6*	-0.03	0.10	0.80

Abbreviations. NHEJ, non-homologous end joining DNA repair. WECARE, The Women's Environmental, Cancer, and Radiation Epidemiology Study; SNP, single nucleotide polymorphism; SE, standard error.
[a] Coefficient of the interaction between radiation dose and SNP, adjusted for age at first diagnosis of breast cancer and three eigenvectors obtained in principal components analysis
[b] Bonferroni-adjusted level of α significance = 7.3×10^{-4}

© 2019 Watt GP et al. *JAMA Network Open.*

eTable 3. Nonhomologous End-Joining Repair Genetic Risk Score, Location-Specific Dose, and Risk of Contralateral Breast Cancer Additionally Adjusting for Variation in Common Breast Cancer Susceptibility Genes in the WECARE Study

NHEJ - GRS	Dose[a] (Gy)	Cases (N)	Controls (N)	RR[b]	95% CI	*P*-value	P_{trend}[c]
Received first breast cancer diagnosis at age <40 years with <5 years after first breast cancer diagnosis[d]							
Low[e] (<75)	0	18	13	Ref	-	-	0.85
	0.01 – <1	7	27	0.3	0.1 – 1.1	0.08	
		16	25	0.9	0.3– 2.7	0.89	
High (≥75)	0	13	12	Ref	-	-	0.51
	0.01 – <1	10	28	0.8	0.2 – 3.0	0.78	
	1+	7	17	1.7	0.5 – 5.8	0.41	
Received first breast cancer diagnosis at age <40 years with ≥5 years after first breast cancer diagnosis							
Low (<75)	0	15	20	Ref	-	-	0.98
	0.01 – <1	15	36	1.2	0.4 – 3.5	0.72	
	1+	18	27	1.0	0.3 – 2.9	0.98	
High (≥75)	0	21	16	Ref	-	-	0.04
	0.01 – <1	15	28	1.1	0.4 – 3.1	0.82	
	1+	17	15	2.9	1.0 – 8.0	0.04	
Received first breast cancer diagnosis at age ≥40 years with <5 years after first breast cancer diagnosis							
Low (<75)	0	65	68	Ref	-	-	0.30
	0.01 – <1	49	145	1.3	0.8 – 2.1	0.33	
	1+	39	110	1.3	0.8 – 2.2	0.36	
High (≥75)	0	76	55	Ref	-	-	0.70
	0.01 – <1	49	100	0.9	0.5 – 1.6	0.79	
	1+	40	78	1.1	0.6 – 2.1	0.67	
Received first breast cancer diagnosis at age ≥40 years with ≥5 years after first breast cancer diagnosis							
Low (<75)	0	108	112	Ref	-	-	0.99
	0.01 – <1	69	150	1.0	0.7 – 1.6	0.91	
	1+	67	139	0.9	0.6 – 1.5	0.79	
High (≥75)	0	107	71	Ref	-	-	0.73
	0.01 – <1	86	110	1.3	0.9 – 2.1	0.21	
	1+	66	112	0.8	0.5 – 1.4	0.45	

Abbreviations. WECARE, The Women's Environmental Cancer and Radiation Epidemiology Study; RR, rate ratio; CI, confidence interval; Gy, gray.

[a] Dose refers to scatter dose to the contralateral breast incurred during treatment for first primary for cases and estimated for the corresponding location (quadrant) of the contralateral breast cancer for controls.

[b] Adjusted for age at first diagnosis of breast cancer, age at menarche, number of full-term pregnancies, first-degree family history of breast cancer, age at menopause, chemotherapy, hormonal therapy, estrogen receptor status of the first primary, progesterone receptor status of the first primary, breast cancer histology, stage, and three eigenvectors obtained in principal components analysis.

[c] *P*-value for test of trend across radiation dose categories

[d] Age- and latency-stratified coefficients are estimated in a single stratified model (N = 3,732)

NHEJ-GRS is divided into two groups based on the median (74) of risk-associated alleles for all participants in the WECARE Study.

© 2019 Watt GP et al. *JAMA Network Open.*

eTable 4. Number of Risk Alleles for Each Gene in the Nonhomologous End-Joining DNA Repair Pathway, Location-Specific Radiation Dose, and Risk of Contralateral Breast Cancer in the WECARE Study Among Women Who Received Their First Breast Cancer Diagnosis When They Were Younger Than 40 Years With a Latency of 5 Years or More

Number of Risk Alleles[a]	Dose[b] (Gy)	CBC (N)	UBC (N)	RR[c]	95% CI	*P*	P_{trend}
**Gene: *PRKDC***							
Below Median (<12)	0	21	20	Ref	-	-	
	0.01 – <1	22	32	1.7	0.7 – 3.9	0.22	0.32
	≥1	21	26	1.5	0.6 – 4.0	0.37	
Above Median (≥12)	0	15	16	Ref	-	-	
	0.01 – <1	8	32	0.9	0.3 – 2.9	0.81	0.14
	≥1	14	16	2.5	0.8 – 8.0	0.12	
**Gene: *XRCC4***							
Below Median (<19)	0	23	22	Ref	-	-	
	0.01 – <1	19	41	1.2	0.5 – 3.0	0.75	0.15
	≥1	23	21	1.9	0.8 – 4.8	0.15	
Above Median (≥19)	0	13	14	Ref	-	-	
	0.01 – <1	11	23	1.5	0.5 – 4.6	0.49	0.21
	≥1	12	21	2.1	0.7 – 6.6	0.20	
**Gene: *XRCC5***							
Below Median (<21)	0	16	19	Ref	-	-	
	0.01 – <1	17	38	1.4	0.5 – 3.6	0.53	0.19
	≥1	20	23	2.0	0.7 – 5.5	0.19	
Above Median (≥21)	0	20	17	Ref	-	-	
	0.01 – <1	13	26	1.3	0.5 – 3.7	0.58	0.16
	≥1	15	19	2.1	0.8 – 5.5	0.15	
**Gene: *XRCC6***							
Below Median (<3)	0	14	7	Ref	-	-	
	0.01 – <1	5	23	0.3	0.1 – 1.4	0.12	0.76
	≥1	15	18	1.0	0.3 – 3.9	0.98	
Above Median (≥3)	0	22	29	Ref	-	-	
	0.01 – <1	25	41	2.2	1.0 – 5.1	0.06	0.05
	≥1	20	24	2.6	1.0 – 6.5	0.04	
**Gene: *DCLRE1C***							
Below Median (<10)	0	17	22	Ref	-	-	
	0.01 – <1	14	32	2.0	0.7 – 5.6	0.20	0.24
	≥1	11	24	1.8	0.6 – 5.3	0.31	
Above Median (≥10)	0	19	14	Ref	-	-	
	0.01 – <1	16	32	1.0	0.4 – 2.6	0.95	0.26
	≥1	24	18	1.6	0.6 – 4.1	0.34	
**Gene: *LIG4***							
Below Median (<8)	0	1	2	Ref	-		
	0.01 – <1	3	5	4.8	0.3 – 88.3	0.29	0.84
	≥1	2	7	1.9	0.1 – 49.2	0.70	
Above Median (≥8)	0	35	34	Ref	-	-	
	0.01 – <1	27	59	1.2	0.6 – 2.5	0.68	0.06
	≥1	33	35	2.0	1.0 – 4.3	0.06	
**Gene: *NHEJ1***							
Below Median (<5)	0	17	18	Ref	-	-	
	0.01 – <1	14	35	6.5	0.3 – 167.0	0.26	0.75
	≥1	19	23	1.6	0.04 – 62.6	0.79	
Above Median (≥5)	0	19	18	Ref	-	-	
	0.01 – <1	16	29	1.2	0.6 – 2.5	0.63	0.07
	≥1	16	19	1.9	0.9 – 3.9	0.09	

© 2019 Watt GP et al. *JAMA Network Open.*

Abbreviations. WECARE, The Women's Environmental Cancer and Radiation Epidemiology Study; RR, rate ratio; CBC, contralateral breast cancer; UBC, unilateral breast cancer; RR, rate ratio; CI, confidence interval

[a] The sum of risk-associated alleles determined by the direction of the main effect of the single nucleotide polymorphism (SNP) in the overall WECARE Study.

[b] Dose refers to scatter dose to the contralateral breast incurred during treatment for the first primary breast cancer, estimated for the corresponding location (quadrant) of the contralateral breast cancer

[c] Adjusted for age at first diagnosis of breast cancer, age at menarche, number of full-term pregnancies, first-degree family history of breast cancer, age at menopause, chemotherapy, hormonal therapy, estrogen receptor status of the first primary breast cancer, progesterone receptor status of the first primary breast cancer, breast cancer histology, stage, and three eigenvectors.

© 2019 Watt GP et al. *JAMA Network Open.*

eFigure. Distribution of Nonhomologous End-Joining Genetic Risk Score in Women with Contralateral Breast Cancer and Women With Unilateral Breast Cancer in the WECARE Study

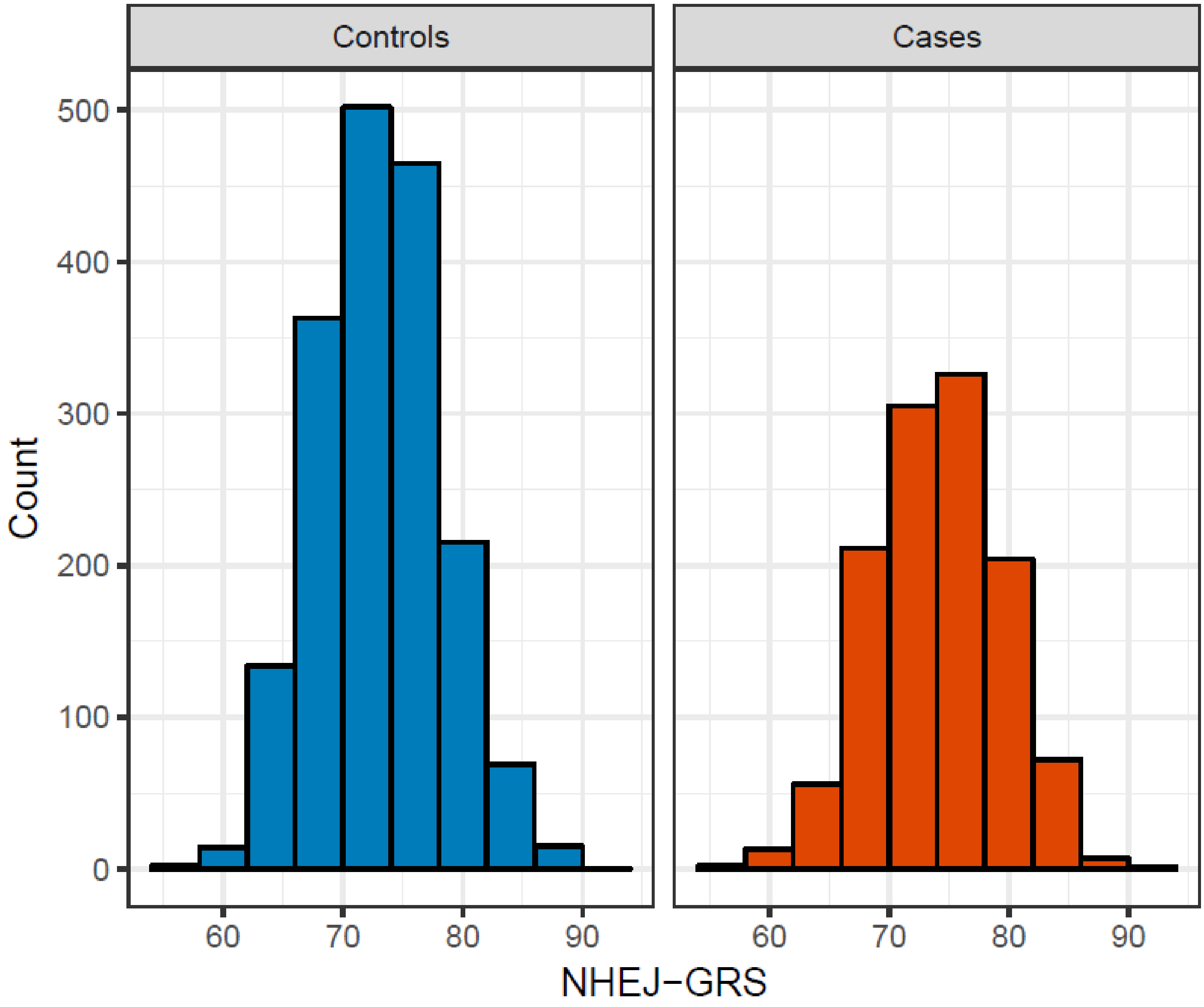

Abbreviation. NHEJ-GRS, Non-Homologous End-Joining DNA Repair - Genetic Risk Score

© 2019 Watt GP et al. *JAMA Network Open.*

References

1. Bernstein JL, Langholz B, Haile RW, et al. Study design: Evaluating gene-environment interactions in the etiology of breast cancer - the WECARE study. Breast Cancer Research 2004;6:R199-R214.

© 2019 Watt GP et al. *JAMA Network Open.*
